# Worms and reproductive failure: First evidence of transplacental *Halicephalobus* transmission leading to repeated equine abortion

**DOI:** 10.1016/j.crpvbd.2025.100309

**Published:** 2025-08-20

**Authors:** Andrea Springer, Christin Krüger, Christina Strube, Dirk Steinhauer

**Affiliations:** a Institute for Parasitology, Centre for Infection Medicine, University of Veterinary Medicine Hannover, Buenteweg 17, 30559, Hanover, Germany; b State Investigation Office Rhineland-Palatinate, Bluecherstrasse 34, 56073, Koblenz, Germany

**Keywords:** Abortion, Cryptic diversity, Free-living nematodes, *Halicephalobus gingivalis*, ITS1, Vertical transmission

## Abstract

Infections with facultatively parasitic *Halicephalobus* spp. nematodes are usually fatal in animals and humans. Here, transplacental transmission of a species of *Halicephalobus* is described for the first time, causing reproductive failure of a mare during two consecutive gestations. In both cases, histology showed adult and larval nematodes in the placenta and various foetal organs, without signs of generalized halicephalobosis in the mare. An identical 18S rRNA-ITS1-5.8S rRNA-ITS2 sequence generated from both placentas showed considerable divergence from a previously sequenced equine isolate, suggesting cryptic diversity among *Halicephalobus* isolates in vertebrates. This ubiquitous nematode may be a cause of equine abortion associated with considerable economic loss. Future research should aim at exploring effective treatment options and clarifying the true taxonomic diversity within the genus *Halicephalobus*.

## Highlights

• Transplacental transmission of *Halicephalobus* documented for the first time.
• Repeated reproductive failure of a mare without further signs of halicephalobosis.
• Disseminated infection in placenta and various foetal organs.
• ITS1 sequence divergence to previous equine isolate suggests cryptic species diversity.

## Graphical abstract

**Figure:**
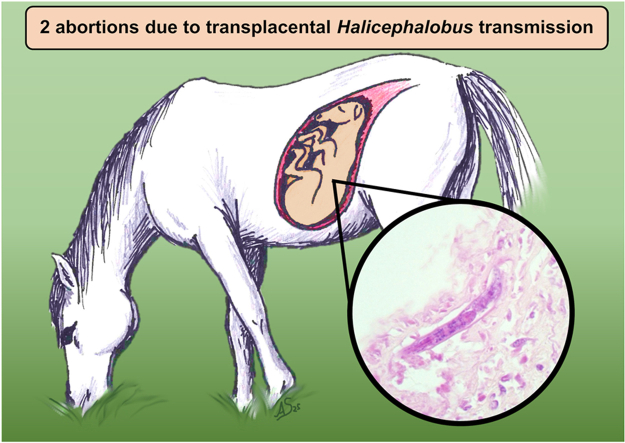

## 1 Introduction

Many infectious agents can cause reproductive failure, including parasites. In horse breeding, pregnancy loss is a major economic concern (Macleay et al., 2022). Infections with the parasitic protozoa *Neospora caninum*, *Neospora hughesi*, *Babesia caballi*, *Theileria equi, Acanthamoeba hatchetii* and *Encephalitozoon cuniculi* are recognized parasitic causes of abortion in horses (Tyrnenopoulou et al., 2021). In contrast, helminth infections are not known to cause equine reproductive failure so far.

Members of the genus *Halicephalobus* (family Panagrolaimidae) are saprophytic nematodes with a worldwide distribution, which occasionally infect vertebrates, predominantly horses (Anderson et al., 1998; Peletto, 2024). To date, all vertebrate infections have been attributed to the species *Halicephalobus gingivalis* (syns. *Halicephalobus deletrix*, *Micronema deletrix*), which shows morphological differences compared to the other seven described species of the genus *Halicephalobus* (Anderson et al., 1998). Besides equine infections, nine human cases (Hoogstraten and Young, 1975; Shadduck et al., 1979; Gardiner et al., 1981; Ondrejka et al., 2010; Papadi et al., 2013; Anwar et al., 2015; Lim et al., 2015; Loo et al., 2015; Monoranu et al., 2015) and a single report from cattle (Enemark et al., 2016) have been published.

The precise route of infection is unknown, but mucosal or skin lesions are presumed entry points for the nematodes (Anderson et al., 1998), followed by haematogenous dissemination (Henneke et al., 2014). In one case, transmammary transmission from a mare to her foal was reported due to a local infection of the mammary gland (Wilkins et al., 2001). Granulomatous inflammation develops in affected tissues, which may contain female nematodes, larvae and eggs, indicating parthenogenetic reproduction (Anderson et al., 1998; Peletto, 2024). In horses as well as humans, the central nervous system is most commonly affected, and rapidly progressing neurological deterioration is a common clinical course (Ondrejka et al., 2010; Peletto, 2024). Besides (meningo)encephalitis, further reported manifestations include nephritis (Schmitz and Chaffin, 2004), orchitis (Pearce et al., 2001), posthitis (Dunn et al., 1993; Muller et al., 2008), mastitis (Wilkins et al., 2001), as well as gingival and mandibular/maxillary granulomas (Anderson et al., 1998; Henneke et al., 2014). In many of these reports, diagnosis was based on the nematodes’ morphology (summarized by Peletto, 2024). Given the lack of molecular data and the fact that the species-discriminating morphological features may be difficult to observe in histological sections (Papadi et al., 2013), it remains unclear if all reported cases were indeed caused by the species *H. gingivalis*. Moreover, phylogenetic analysis of four clinical and two free-living strains revealed that genetically diverse isolates can cause vertebrate infections (Nadler et al., 2003).

Most equine and all reported human infections were diagnosed post-mortem. Successful treatment has only been reported in three equine cases with localised manifestations that resolved after surgical removal and/or systemic anthelminthic treatment (Dunn et al., 1993; Pearce et al., 2001; Schmitz and Chaffin, 2004). In other cases, progression from localised infection to fatal neurological involvement occurred despite anthelminthic treatment (Ferguson et al., 2008; Henneke et al., 2014). In fact, an *in vitro* study suggests a high level of ivermectin and thiabendazole tolerance in clinical as well as free-living isolates (Fonderie et al., 2012). Here, transplacental transmission of a *Halicephalobus* sp. in the equine host is described for the first time, resulting in reproductive failure during two consecutive gestations.

## 2 Materials and methods

### 2.1 Case description

A Connemara mare, born in 2017, was imported from Ireland to Germany together with a healthy foal in 2021. She joined a stud farm with approximately 30 animals, including a stallion for natural mating. Routine deworming on the farm was performed two to four times per year, depending on the animals’ age group, by use of ivermectin or fenbendazole, and praziquantel. The mare gave birth to healthy foals in 2022 and 2023, before a stillbirth of a mature foal occurred in April 2024. The stillborn foal and its placenta were subjected to a pathological examination.

After recovering from the stillbirth, the mare showed no abnormal clinical signs. Anthelminthic treatment was attempted with fenbendazole at a dose of 50 mg/kg body weight daily for three consecutive days in May 2024. After resuming a normal oestrous cycle, she was mated again in July 2024 but aborted at the end of November. The foetus and placenta were again subjected to a pathological examination.

### 2.2 Molecular analyses

For molecular diagnosis, DNA was isolated from several tissue samples. In the first case, four tissue samples from the placenta of the stillborn foal were processed *via* the NucleoSpin Tissue Kit (Macherey-Nagel GmbH, Dueren, Germany) according to the manufacturer’s instructions. As PCR amplification of *Halicephalobus* sp. DNA was not successful from these samples, six further placenta samples were incubated in 180 μl DirectPCR® cell lysis reagent (Peqlab, Erlangen, Germany) supplemented with 20 μl proteinase K at 55 °C overnight, followed by 85 °C for 45 min. The resulting lysate was diluted 1:10 for use in PCR reactions. In the second case, three tissue samples each of the placenta and foetal liver, lungs, kidneys, spleen and intestine were processed *via* the DirectPCR® cell lysis reagent as described above, and three further placenta samples *via* the NucleoSpin Tissue Kit.

The 18S ribosomal RNA (rRNA)-internal transcribed spacer (ITS) 1-5.8S rRNA-ITS2 region was targeted using the primers 652 (Callejón et al., 2013) and NC2 (Gasser et al., 1996). Amplification was achieved with DreamTaq® DNA polymerase (5 U/μl, Thermo Fisher Scientific Inc., Schwerte, Germany) in a 25 μl reaction set-up with 2 μl template. Reaction conditions followed the polymerase manufacturer’s instructions, with an annealing temperature of 50 °C. PCR products were custom Sanger sequenced (Microsynth Seqlab Laboratories, Göttingen, Germany) and compared to publicly available sequences using NCBI Blast.

## 3 Results

### 3.1 Pathological examination

**Fig. 1:**
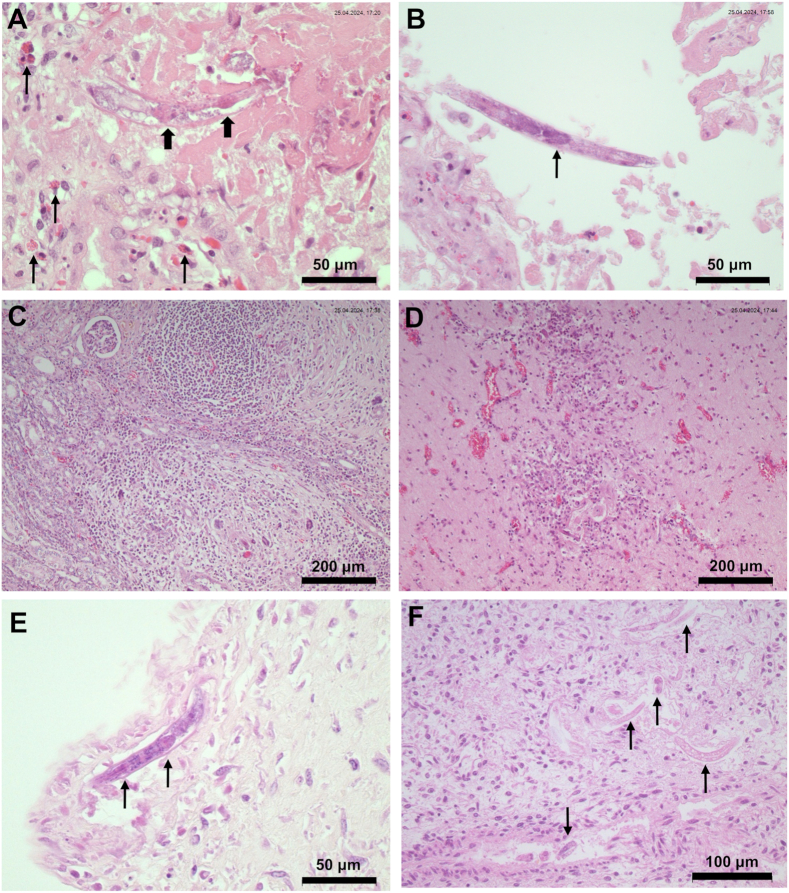
Histological sections (haematoxylin-eosin staining) from different tissues of foal 1 (**A**–**D**) and foal 2 (**E**–**F**). **A** Adult nematode with a rhabditiform oesophagus (*thick arrows*) and presence of eosinophilic granulocytes (*slender arrows*) within necrotic lesion of the placenta. **B** Section of adult female nematode in the placenta, showing the dorsally flexed ovary (*arrow*). **C** Granulomatous lesions in the kidney. **D** Granulomatous lesions in the brain. **E** Adult nematode with a rhabiditiform oesophagus (*arrows*) in the placenta. **F** Multiple nematodes (*arrows*) in the lung. The downwards arrow indicates an embryonated egg inside a blood vessel.

The placenta of the stillborn foal was thickened, dark red with multiple yellow-grey patches and a brown exsudate. By histopathology, adult female nematodes characterized by a rhabditiform oesophagus (Fig. 1A) and dorsally flexed ovary (Fig. 1B) as well as larval nematode stages and eosinophilic granulocytes were demonstrated within necrotic lesions of the placenta. Furthermore, multifocal granulomatous lesions with nematode stages and eosinophils were present in the foal’s kidneys (Fig. 1C) and central nervous system (Fig. 1D).

The second foetus showed signs of autolysis when received for pathological examination. Histopathology of the placenta as well as the foetal liver and lungs showed multifocal necrosis with intralesional nematode stages (Fig. 1E–F). Nematode stages were also observed within blood vessels (Fig. 1F). Other organs were not subjected to histopathology due to their autolytic state.

### 3.2 Sequencing results

**Fig. 2:**
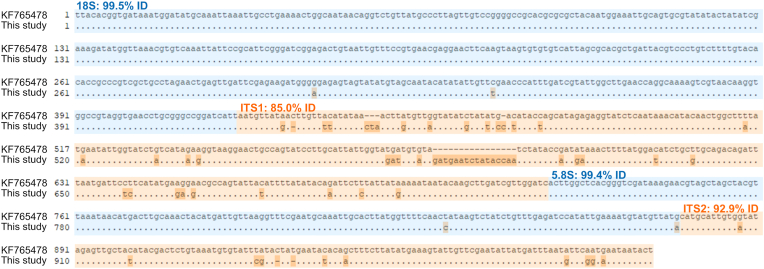
Alignment of the *Halicephalobus* sp. 18S rRNA-ITS1-5.8S-ITS2 sequence obtained in the present study with the only publicly available sequence of comparable length (GenBank: KF65478). Matching bases are represented by dots, while a dash indicates a gap. The partial 18S rRNA and 5.8S rRNA sequences are highlighted in blue, while the ITS sequences are highlighted in orange.

Two samples, one each from the placenta of the first and the second foal, yielded a *Halicephalobus* sp. sequence, whereas PCR amplification from the remaining tissue samples was not successful or yielded bacterial sequences. The two obtained *Halicephalobus* sp. sequences of approximately 1000 bp (GenBank accession nos. PV917188 and PV917189) were 100% identical. For comparison across the entire sequence length, only a single *Halicephalobus* sequence (*H. gingivalis* isolate “Indigo S1342-13”, from a horse in Germany, GenBank: KF765478) was publicly available, showing an overall 94% nucleotide identity (100% query cover) to the present isolate (Fig. 2). Considering only the partial 18S rRNA sequence (421 bp), > 99% nucleotide identity with “Indigo S1342-13” as well as various morphologically characterized and cultured *H. gingivalis* isolates (Nadler et al., 2003) was observed (97% query cover, e.g. GenBank: MK087059 and HQ697250). Regarding the ITS1 and partial ITS2 regions, nucleotide identity with isolate “Indigo S1342-13” amounted to 85.0% and 92.9%, respectively (100% query cover each, Fig. 2). For ITS1, two further partial sequences assigned to *Halicephalobus* spp. (GenBank: MT310715 and MT310716) were available in GenBank, but showed even lower identity (79.8% and 79.1% with a query cover of 78% and 37%, respectively).

## 4 Discussion

To the authors’ knowledge, this is the first report of prenatal *Halicephalobus* spp. infection, and the first report of helminths as a cause of equine abortion in general. However, *Halicephalobus* sp. infection was previously reported in two foals born subsequently to a mare, with clinical signs starting at 18 days and 7 weeks of age, respectively, whereby the route of infection was suspected to be prenatal, perinatal or transmammary (Spalding et al., 1990). Moreover, a case of probable transmammary transmission from a mare with localized *Halicephalobus* sp. infection of the mammary gland to her foal was reported (Wilkins et al., 2001). In that latter case, the mare developed fatal neurological involvement more than a year after the initial diagnosis of verminous mastitis. Similarly, the present case suggests that localized *Halicephalobus* sp. infection can persist for months without central nervous system involvement. Although reinfection during the second gestation remains a theoretical possibility, this seems rather unlikely due to the general rarity of cases, and the reported ineffectiveness of anthelminthics. Here, treatment with fenbendazole was attempted, but seemed unsuccessful, as reported previously (Henneke et al., 2014). In other reports, repeated applications of high-dose ivermectin (1.2 mg/kg) (Ferguson et al., 2008) or moxidectin (0.4 mg/kg) (Muller et al., 2008) were also ineffective. To avoid further reproductive problems, the mare was taken out of breeding. Consultation with the owner indicated that 7 months after the abortion, she remained clinically healthy. However, as no known effective treatment exists, it is possible that dissemination may eventually occur. The owner reported that another mare from the same farm was euthanized due to progressing neurological deficits, including blindness and circling movements, during 2023. However, as this animal was not subjected to a post-mortem examination, involvement of *Halicephalobus* sp. remains speculative. Since the infection is rarely diagnosed ante-mortem, knowledge on the pathogenesis is limited and factors favouring dissemination are unknown. In most cases, the affected individuals had no history of immunosuppression (Lim et al., 2015), although immunosuppressive treatment administered upon onset of neurological symptoms might impact the clinical course (Monoranu et al., 2015).

In many previous cases, diagnosis of the species *H. gingivalis* was based solely on morphology (summarized by Peletto, 2024), and molecular data are limited. In the present case, identical 18S rRNA-ITS1-5.8S rRNA-ITS2 sequences were obtained from the placenta of both foals. Regarding the partial (421 bp) 18S rRNA gene, > 99% nucleotide identity with various morphologically characterized and cultured *Halicephalobus* spp. isolates, e.g. “SAN100” obtained from a horse in Canada, “JB043” from potting soil in Germany and “JB128” from compost in the USA (Nadler et al., 2003), was observed. However, the 18S rRNA gene has little sequence variation and thus low species discriminatory power in several nematodes (Callejón et al., 2013). Regarding ITS1, only one complete and two partial *Halicephalobus* spp. sequences were publicly available for comparison and showed ≤ 85% nucleotide identity to the present isolate. Therefore, the genetic data underline the possible existence of cryptic species within the morphospecies *H. gingivalis*, as previously proposed by Nadler et al. (2003) based on sequence variation in the 28S rRNA gene of four horse-derived and two free-living isolates, which did not show any morphological differences. On the other hand, considerable intraspecific sequence variation in the ITS1 region has been shown for some plant-parasitic nematodes, e.g. up to 13% for certain *Cephalenchus* spp. (Pereira and Baldwin, 2016). Intriguingly, this high intraspecific divergence was primarily due to intragenomic polymorphisms within, rather than among individuals (Pereira and Baldwin, 2016). In contrast, the fact that two identical *Halicephalobus* sp. sequences were derived in the present study argues against intragenomic polymorphisms in this case. Further molecular studies are thus necessary to clarify the true taxonomic diversity within the genus *Halicephalobus*.

## 5 Conclusions

Although considered rare, infections with *Halicephalobus* spp. may have detrimental consequences and pose a diagnostic and therapeutic challenge. As horses are valuable animals, the economic loss can be considerable. Besides representing a differential diagnosis for neurological deterioration in animals and humans, the present case showed that *Halicephalobus* spp. infection also needs to be considered as a cause of equine abortion. The fact that the mare remained otherwise asymptomatic indicates that *Halicephalobus* spp. infections may occur more frequently than currently assumed. This is especially concerning in light of the worldwide, ubiquitous distribution of this facultative parasite.

## Ethical approval

As only abortion material was investigated, no ethical approval was required.

## CRediT authorship contribution statement

**Andrea Springer:** Investigation, Visualization, Writing – original draft, Writing – review & editing. **Christin Krüger:** Investigation, Visualization, Writing – review & editing. **Christina Strube:** Supervision, Writing – review & editing. **Dirk Steinhauer:** Investigation, Visualization, Supervision, Writing – review & editing.

## Funding

This research did not receive any specific grant from funding agencies in the public, commercial, or not-for-profit sectors.

## Declaration of competing interests

The authors declare that they have no known competing financial interests or personal relationships that could have appeared to influence the work reported in this paper.

## Data Availability

Data supporting reported results is contained within the article. The newly generated sequences were deposited in the GenBank database under the accession numbers PV917188-PV917189.
